# The Memory Inflation Response Against Spread‐Defective CMV Requires Priming‐Independent CD4^+^ T Cell Help

**DOI:** 10.1002/eji.70225

**Published:** 2026-06-17

**Authors:** Jack Barton, Yeonsu Kim, Matthias T. Warkotsch, Luka Cicin‐Sain, Mateusz P. Poltorak, Veit R. Buchholz, Dirk H. Busch

**Affiliations:** ^1^ Institute For Medical Microbiology Immunology and Hygiene TUM School of Medicine and Health Technical University of Munich (TUM) Munich Germany; ^2^ Department of Viral Immunology Helmholtz Centre for Infection Research Braunschweig Germany; ^3^ Centre for Individualized Infection Medicine a Joint Venture of the Medical School Hannover and the Helmholtz Centre For Infection Research Hannover Germany; ^4^ German Center for Infection Research (DZIF) Partner Site Munich Hannover‐Braunschweig Germany; ^5^ Division of Immunology German Cancer Research Center (DKFZ) Heidelberg Germany; ^6^ German Center for Infection Research (DZIF) Partner Site Munich Munich Germany

**Keywords:** antigen, biology, CD8, cytotoxic T cell, effector, immunology, T cell, vaccination, virus

## Abstract

Memory inflation (MI) is a distinctive CD8^+^ T cell response to chronic cytomegalovirus (CMV) infection, characterized by non‐contracting, functional populations that are predominantly effector differentiated. Harnessing such responses has received growing attention as a promising vaccination strategy, using spread‐defective CMV‐vectors. However, many underlying mechanistic aspects of CD8^+^ MI responses, including their dependency on CD4^+^ T cell help, remain unclear. Using a Rag KO transfer model, we demonstrate that CD4^+^ T cells enhance effector differentiation of inflationary CD8^+^ T cells when present during priming, or when introduced at late timepoints post‐infection, indicating a continuous, priming‐independent support mechanism. This helper requirement was specific to spread‐defective CMV infection, whereas spread‐competent virus induced MI even in the absence of CD4^+^ T cells. We confirmed the importance of continuous support in wild‐type mice, where late CD4 depletion attenuated an established MI response. Gene set enrichment analysis implicates IFNγ, TNF, and IL‐1 signaling as potential mediators of CD4^+^ T cell support. Our findings clarify CD4^+^ T cell help requirements for maintaining effector populations in MI during chronic antigen exposure. These results have particular relevance for spread‐defective CMV‐based vaccination strategies and T cell‐based therapies in which the persistence and expansion characteristic of MI are desirable qualities.

## Graphical abstract

Inducing memory inflation (MI) is a promising future vaccination strategy. We show that spread‐defective cytomegalovirus does not induce MI in the absence of CD4^+^ T cells. Support from CD4^+^ T cells can drive effector differentiation of MI cells, even late in the response, and contributes to long‐term response maintenance.

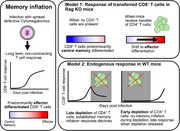

## 1 Introduction

Cytotoxic CD8^+^ T cell responses constitute an important part of adaptive immunity, enabling control of intracellular pathogens such as viruses, which are often inaccessible to antibody control. The classical CD8^+^ T cell response is characterized by an initial population expansion in response to antigenic stimulation, followed by a sharp population contraction of effector T cells upon resolution of the pathogenic insult [1]. A residual population of memory T cells persists afterward, which is able to rapidly proliferate and respond to further pathogenic stimulation [2].

However, if cognate antigen is continuously available for a prolonged period, as in the case of chronic infection or tumor disease, responding CD8^+^ T cells can lose functionality and enter a state of exhaustion. This renders them unable to proliferate and produce cytokines in response to further stimulation [3]. Exhaustion remains a critical problem for T cell‐based immunotherapies. A curious exception to this limitation of T cell biology can be found in the response to the betaherpesvirus cytomegalovirus (CMV). CMV establishes a lifelong infection in its host, and throughout the course of this infection, sporadically reactivates in latently infected cells, thereby presenting viral antigens on a chronic time scale [4]. This chronic stimulation drives an unusual CD8^+^ T cell response known as memory inflation (MI), defined by three key features: (1) maintenance of memory cell populations with restricted or no contraction, (2) a predominantly effector memory phenotype, and (3) sustained functionality without exhaustion [5, 6, 7, 8]. Harnessing the properties of the inflationary response is an attractive prospect in contexts where large functional T cell populations are required, such as tumor disease, where persistence of the CAR T cell product is correlated with patient response [9]. Additionally, generation of MI responses with a CMV‐based vaccination vector has shown promising results in controlling Simian Immunodeficiency Virus infection in primates [10].

An unresolved question in MI is the involvement of CD4^+^ T cells. In classical CD8^+^ T cell memory responses, the importance of CD4^+^ T cell help is well established [11]. CD4^+^ T cells confer improved survival and functionality on CD8^+^ T cells throughout initial priming [12], resolution [13], and recall [14]. MI similarly exhibits CD4^+^ T cell dependence in murine models of CMV [15] and adenoviral vector infection [16]. However, CD4^+^ T cell dependence in MI exhibits substantial context‐dependent variation, a pattern that underscores our limited understanding of how this support is mediated. The requirement for CD4^+^ T cell help varies considerably across viral epitopes: while the inflationary response to the IE3 epitope is completely abolished in the absence of CD4^+^ T cells, the m139 response is only partially reduced, and the M38 response appears unaffected. This dependence also varies with viral biology, as the M38 inflationary response becomes CD4^+^ T cell‐dependent when CMV replication is pharmacologically controlled [17], though whether this extends to spread‐defective viruses remains untested.

Previous investigations into the mechanisms of CD4^+^ T cell support in MI have primarily employed genetic knockout models, which have inherent limitations. CD4 or MHCII knockout mice offer complete ablation of the CD4^+^ T cell compartment but do not permit more nuanced manipulations such as modulating antigen specificity, population size, or timing of depletion. Meanwhile, genetic knockout models have also been used to determine specific cytokines relevant to chronic T cell responses, but have so far not established clear mediators of CD4^+^ T cell support in MI. IL21‐R knockout, for example, prevents the development and persistence of large virus‐specific CD8^+^ T cell populations in chronic lymphocytic choriomeningitis virus (LCMV) infection [18], while interestingly, IL‐21 is also required for the MI response to adenoviral vector vaccination [19]. However, the MI in response to MCMV is apparently independent of IL‐21 [17]. In this same study, the role of IL‐2 was also investigated, showing that IL‐2R knockout CD8^+^ T cells failed to generate M38 responses, although whether this effect is mediated through CD4^+^ T cells was not established. In this study, we aimed to clarify how CD4^+^ T cells facilitate MI responses to a murine spread‐defective CMV lacking a glycoprotein required for cell entry, a model reflecting CMV modification in a vaccination context [20]. We show that in the absence of CD4^+^ T cells, MI fails to generate not only in terms of expansion, but also in effector differentiation, a defining feature of MI. This failed MI response is nonetheless distinct from responses with classical kinetics. We show that this effector differentiation can be restored by the addition of CD4^+^ T cells at early or late timepoints, demonstrating a priming‐independent mechanism of support. By depleting CD4^+^ cells in WT mice, we show that continuous CD4^+^ T cell support is important for maintenance of the MI response. Finally, we performed single‐cell RNA sequencing of CMV‐reactive CD8^+^ T cells to comprehensively characterize transcriptional differences between these cells in the presence or absence of CD4^+^ T cell support. Gene set enrichment analysis was then used to infer potential cytokines that may confer helper T cell support. Together, these data implicate a novel mechanism of CD4^+^ T cell support of CD8^+^ MI.

## 2 Results

### 2.1 Spread‐Defective Murine CMV Fails to Induce Effector Differentiated MI Response in Rag KO Mice

**FIGURE 1:**
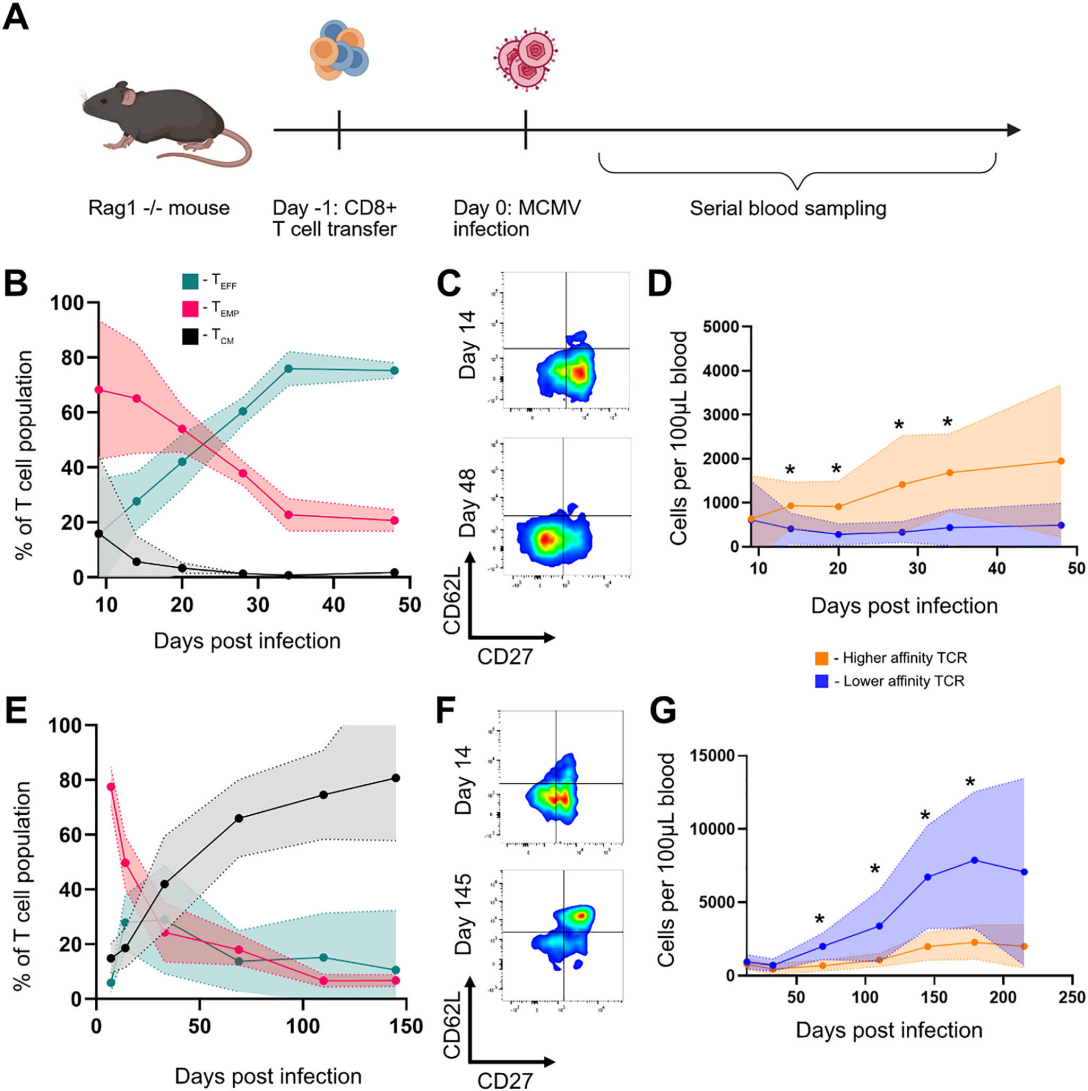
(A) Experimental overview. Rag KO mice received a transfer of two CD8^+^ T cell populations from retrogenic mice, distinguished from one another by fluorescent reporter molecules. These T cells are specific for the model epitope SIINFEKL, one population with a higher affinity receptor, and one with a lower affinity receptor. The following day, mice were infected with murine cytomegalovirus expressing SIINFEKL on the inflation associated gene IE2. T cell responses were monitored by longitudinal blood sampling. (B) T cell effector differentiation over time in response to host infection by spread competent MCMV‐IE2‐SIINFEKL infection (500 PFU). Typical of memory inflation responses, T effector cells became predominant in the population. (C) Exemplary FACS plots showing greater T cell effector differentiation over time (day 14 vs. day 48). Differentiation defined by CD27 and CD62L expression: effector memory—CD27^−^CD62L^−^, T effector memory precursor—CD27^+^CD62L^−^, central memory—CD27^+^CD62L^+^. (D) T cell counts in peripheral blood over time, spread competent virus infection. The higher affinity T cell population is numerically dominant. Typical of memory inflation, populations do not contract, but rather maintain at a stable level or gradually expand. (E) T cell differentiation over time in response to host infection by spread‐defective MCMV‐ΔgL‐IE2‐SIINFEKL (1 × 10^5^ PFU), showing a lack of effector differentiation, and predominance of CM cells over time. Exemplary FACS plots in (F). (G) T cell counts in peripheral blood over time, spread‐defective virus infection. Lower affinity population is numerically dominant. Panels (B, C, E, F) show data from the higher affinity T cell population, and the lower affinity population showed comparable differentiation responses (Figure S1). Graphs in (B, D, E, G) show mean with standard deviation indicated by shaded area. Experiment conducted with 11–13 mice. Comparisons in (D and G) between cell populations at each timepoint were performed using two‐sided paired *t*‐tests with Benjamini–Hochberg correction for multiple comparisons across timepoints. *p < 0.05.

MI responses against certain epitopes depend on CD4^+^ T cell help [15]; however, the underlying cause of this dependence is not understood. To investigate the mechanisms underlying CD4^+^ T cell support of MI and characterize failed inflationary responses in the absence of CD4^+^ T cell help, we employed a Rag KO transfer system in which defined CD8^+^ T cell populations are transferred into lymphopenic mice (Figure 1A). Such a system allows us to study individual CD8^+^ T cell CMV responses in isolation, and with complete absence of T helper cells, by selecting CD8^+^ T cells specific for a desired CMV epitope. To study a response in greater detail, a further layer of resolution can be applied by transferring T cells with differential epitope affinity, which can then be monitored at a clonal level with fluorescent reporters and congenic markers [21].

We transferred SIINFEKL‐specific CD8^+^ T cells (a high‐ and a low‐affinity population, distinguished by fluorescent reporters, Figure S1A) into Rag KO mice, which were subsequently infected with murine cytomegalovirus (MCMV) expressing SIINFEKL on the c‐terminus of the IE2 protein, which typically results in inflationary CD8^+^ T cell responses [22, 23]. This system produced a SIINFEKL‐specific inflationary response that developed into a predominantly effector memory population, typical of MI (Figure 1B, C, Figure S1B). Consistent with expected affinity‐related dynamics, the higher‐affinity population established numerical dominance at early timepoints (Figure 1D), with both populations maintaining steady or slightly increasing numbers thereafter.

The sensitivity of MI to the absence of CD4^+^ T cell support can be enhanced by pharmacologically controlling viral activity [17]. MI responses to vaccine vectors, controlled by modification to impede viral spread, might similarly be especially dependent on CD4^+^ T cell support, although this is currently unexplored. We repeated our experimental setup using a spread‐defective virus, MCMV‐ΔgL‐IE2‐SIINFEKL, which lacks glycoprotein L required for cell entry. Although this spread‐defective (single cycle) virus can induce MI in wild‐type C57BL/6 mice [24], CD8^+^ T cells in the Rag KO transfer system became predominantly central memory differentiated (Figure 1E, F, Figure S1C), contrary to the typical MI phenotype. Despite this, the other features of MI were retained, as transferred CD8^+^ T cells remained functional (Figure S2), and continued to expand over the course of the experiment (Figure 1G). We therefore considered whether this was a “central memory cell inflation” driven by persistent antigen exposure, which somehow failed to induce effector differentiation, or alternatively, merely homeostatic proliferation in the lymphocyte‐deficient Rag KO system. To distinguish these possibilities, we compared T cell responses between MCMV‐ΔgL‐infected mice and mice receiving acute stimulation with Modified Vaccinia Ankara expressing SIINFEKL (MVA‐Ova) (Figure S3A). In both groups, transferred T cells formed predominantly central memory populations with continuous expansion, confirming the relevance of antigen‐independent, homeostatic proliferation (Figure S3B). However, despite initial similarity in population sizes between groups, MCMV‐infected mice exhibited a trend toward greater long‐term expansion of the central memory compartment, although this did not reach statistical significance (Figure S3C,D).

### 2.2 CD4^+^ T Cells Rescue Effector Differentiation in Spread‐defective Infection

**FIGURE 2:**
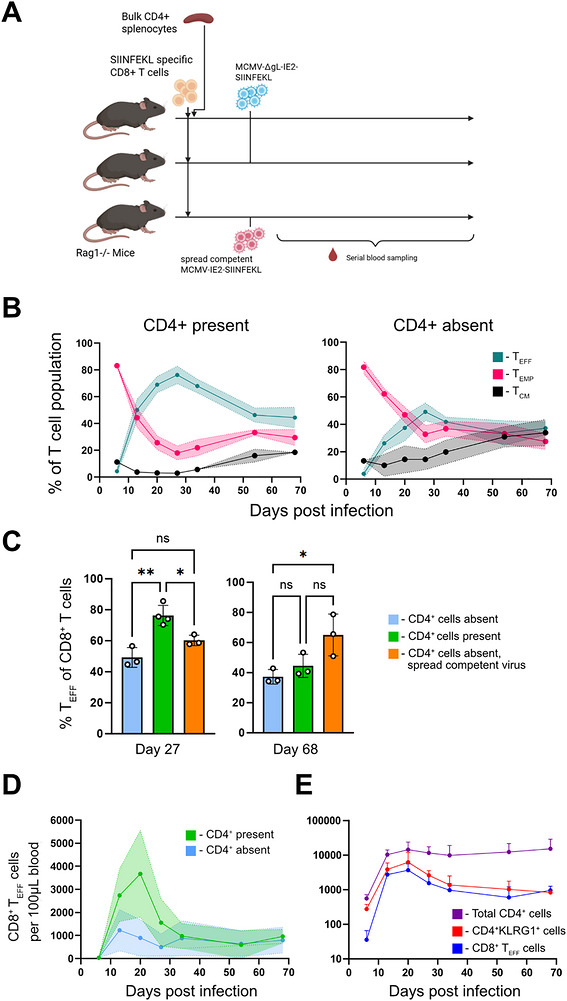
(A) Experimental overview. Rag KO mice received a transfer of SIINFEKL‐specific T cells from retrogenic mice. One group additionally received a bulk transfer of 1.2 × 10^6^ CD4^+^ splenocytes the same day. The following day, mice were then infected with spread‐defective MCMV‐ΔgL‐IE2‐SIINFEKL (1 × 10^5^ PFU). A third group received no CD4^+^ splenocytes and was infected with spread competent MCMV‐IE2‐SIINFEKL (500PFU). T cell responses were monitored by longitudinal blood sampling. (B) CD8^+^ T cell differentiation over time after infection with MCMV‐ΔgL‐IE2‐SIINFEKL, in mice where CD4^+^ T cells are present (left) or absent (right). Differentiation defined by CD27 and CD62L expression: effector memory—CD27^−^CD62L^−^, T effector memory precursor—CD27^+^CD62L^−^, central memory—CD27^+^CD62L^+^. (C) CD8^+^ T cell effector differentiation in MCMV‐ΔgL‐IE2‐SIINFEKL infected mice was seen to peak at day 27 post infection. At this timepoint, effector differentiation was greater in mice with CD4^+^ T cells than in mice absent of CD4^+^ T cells. By day 68, this difference is gone, and CD8^+^ T cells in mice infected with spread‐competent virus are most effector differentiated. (D) Absolute counts of CD8^+^ T cells in spread‐defective virus‐infected mice, peripheral blood. In the presence of CD4^+^ T cells, the CD8^+^ T cell population increases, but rapidly contracts to levels comparable to CD4^+^ T cell‐absent mice. (E) Absolute counts of total CD4^+^ T cells in blood are stable over time. The kinetics of the CD4^+^KLRG1^+^ population however closely match that of the CD8^+^ T effector population. Graphs in (B, D, E) show mean with standard deviation indicated by shaded area or error bar. Experiment conducted with three to four mice per group. Statistical test in (C) is one‐way ANOVA with Tukey's post hoc test. *p < 0.05, **p < 0.01. Groups in (D) were evaluated by Welch's *t*‐tests at each timepoint with Benjamin–Hochberg correction for multiple comparisons, *p *> 0.05 at all timepoints.

Next, we investigated whether CD4^+^ T cells could rescue effector differentiation of inflationary cells in our system. Rag KO mice received SIINFEKL‐specific CD8^+^ T cells followed by infection with spread‐defective MCMV‐ΔgL (Figure 2A). One group additionally received 1.2 × 10^6^ CD4^+^ T cells each from wild‐type C57BL/6 spleens, while a third group received no CD4^+^ T cell transfer and was infected with spread‐competent MCMV. In spread‐defective MCMV‐infected mice that had not received CD4^+^ T cells, effector differentiation of CD8^+^ T cells was limited, as we had observed previously (Figure 1E). Interestingly, CD4^+^ T cell recipients showed a clear increase in CD8^+^ T cell effector differentiation, most apparent at a peak in the proportion of effector differentiated CD8^+^ T cells at day 27 (Figure 2B, C). However, this effect was transient, with effector differentiation between the two groups becoming comparable by the last point in our observation period at day 68, while the central memory proportion increased over time in both groups. Absolute counts revealed that this phenotypic evolution resulted from an initially enhanced expansion of the CD8^+^ effector T cell population in CD4^+^ T cell recipients (Figure 2D), which contracted after peaking around day 20 post‐infection, although the difference at this peak did not reach statistical significance. As previously observed, effector differentiation was maintained in mice infected with spread competent virus, despite the lack of CD4^+^ T cells (Figure 2C). Total CD4^+^ T cell populations remained stable following initial expansion (Figure 2E), though interestingly, the CD4^+^KLRG1^+^ population kinetics closely paralleled those of the CD8^+^ effector T cell population.

This rescue of effector differentiation by CD4^+^ T cell transfer aligns with previous reports of CD4^+^ T cell support for MI. However, the transient nature of this effect suggests that continuous support is required to maintain MI, rather than T cell help being required only for CD8^+^ T cell programming during priming.

### 2.3 CD4^+^ T Cells Support Effector Differentiation Independently of Priming

**FIGURE 3:**
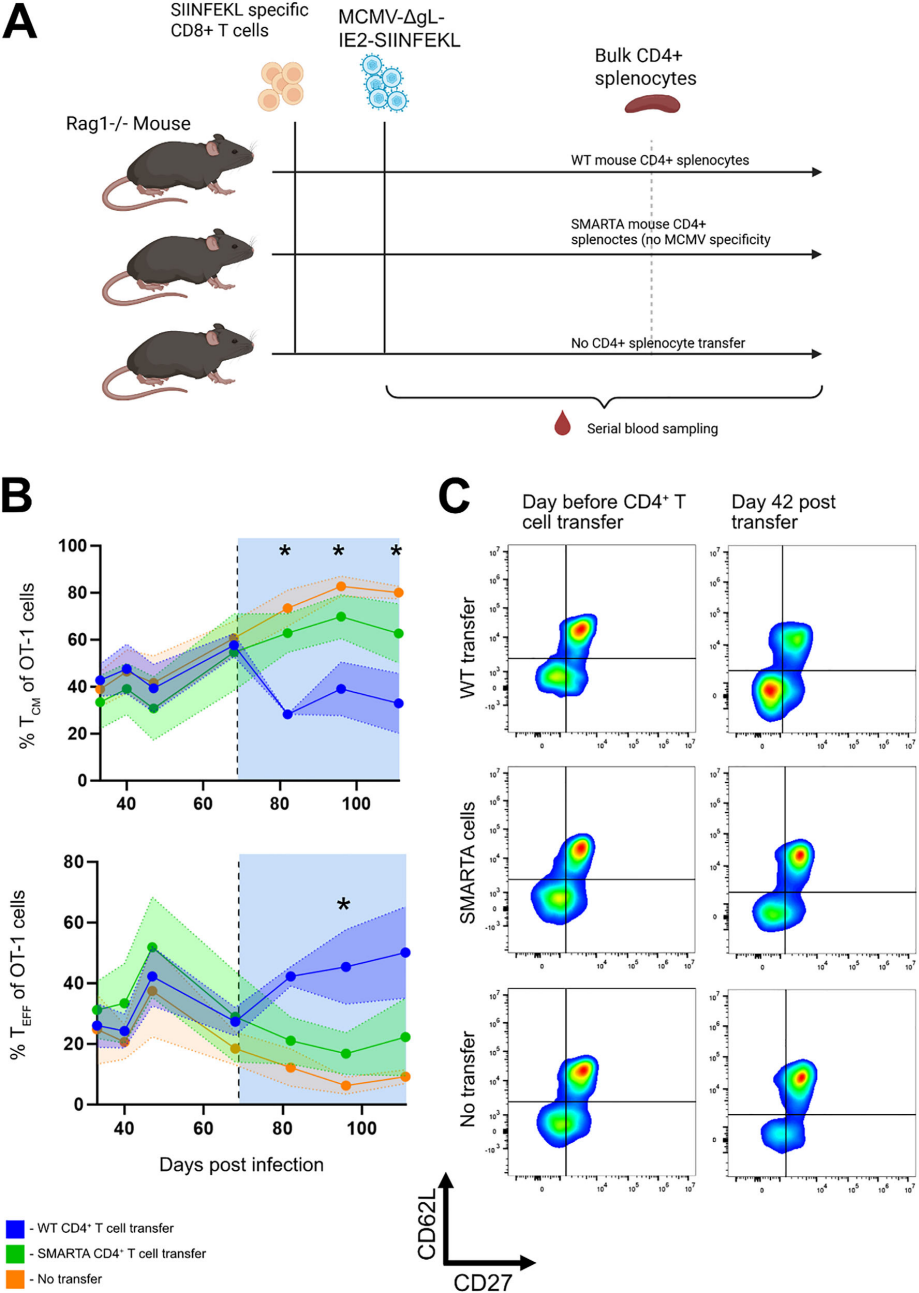
(A) Experimental overview. Rag KO mice received SIINFEKL‐specific T cells from OT‐I mice. The following day, mice were infected with spread‐defective MCMV‐ΔgL‐IE2‐SIINFEKL. At a late timepoint, one group of mice receives a transfer of 3 × 10^6^ CD4^+^ T cells from a C57Bl/6 mouse, while another group receives non‐MCMV‐reactive CD4^+^ T cells from a SMARTA mouse, and a third group receives no CD4^+^ T cells. Three mice per group. (B) Proportion of transferred CD8^+^ T cells with central memory phenotype (CD27^+^CD62L^+^) over time (top), and effector memory phenotype (CD27^−^CD62L^−^) over time (bottom). Light blue shading indicates time after CD4^+^ T cell transfer. In mice receiving C57Bl/6 WT CD4^+^ T cells, a clear shift from central memory to effector memory phenotype is seen after this transfer. (C) Exemplary FACS plots showing effector differentiation of CD8^+^ T cells immediately before CD4^+^ T cell transfer (left), where central memory cells form the largest fraction in all groups. At the experimental endpoint, 42 days post transfer (right), central memory cells are the main constituent of the CD8^+^ T cell population in mice with no CD4^+^ T cell transfer, and mice receiving SMARTA cells, while in mice receiving C57Bl/6 CD4^+^ T cells, the CD8^+^ T cell population is now predominantly effector memory. Graphs show mean with standard deviation indicated by shaded area. Statistical significance (*p < 0.05) was determined in a comparison between WT CD4^+^ T cell recipient and no transfer groups, using Welch's *t*‐tests at each timepoint with Benjamini‐Hochberg correction for multiple comparisons.

To confirm whether CD4^+^ T cells can mediate effector differentiation support through a priming‐independent mechanism, we transferred SIINFEKL‐specific OT‐I CD8^+^ T cells into Rag KO mice and infected them with spread‐defective MCMV (Figure 3A). Then, at a late timepoint after OT‐I cells had predominantly formed central memory populations in all groups (Figure 3B, C), we administered 3 × 10^6^ CD4^+^ T cells. One group received cells from wild‐type C57BL/6 mice, which probabilistically include MCMV‐reactive cells within a naturally diverse repertoire. Another group received CD4^+^ T cells from SMARTA mice expressing TCRs specific for the LCMV peptide gp33, which is not present in MCMV, and therefore are unlikely to be reactive to MCMV. CD8^+^ T cells in mice receiving wild‐type CD4^+^ T cells showed decreased central memory differentiation, and increased CD8^+^ T cell effector differentiation (Figure 3B, C, Figure S4). This shift from central memory phenotype to effector memory was sustained, and the emergent difference in the central memory proportion of CD8^+^ T cells remained statistically significant compared to the CD4^+^ T cell‐absent group until the experimental endpoint 42 days post‐CD4^+^ T cell transfer. Mice receiving SMARTA cells showed ambiguous results, with no statistically significant difference in effector differentiation compared to either the WT transfer group or the CD4^+^ T cell absent group. Therefore, while CD4^+^ T cell support can enhance effector differentiation of MI cells, whether this support requires CMV‐specific CD4^+^ T cells remains unclear.

### 2.4 CD4^+^ T Cells Continuously Support MI in Wild‐Type Mice

**FIGURE 4:**
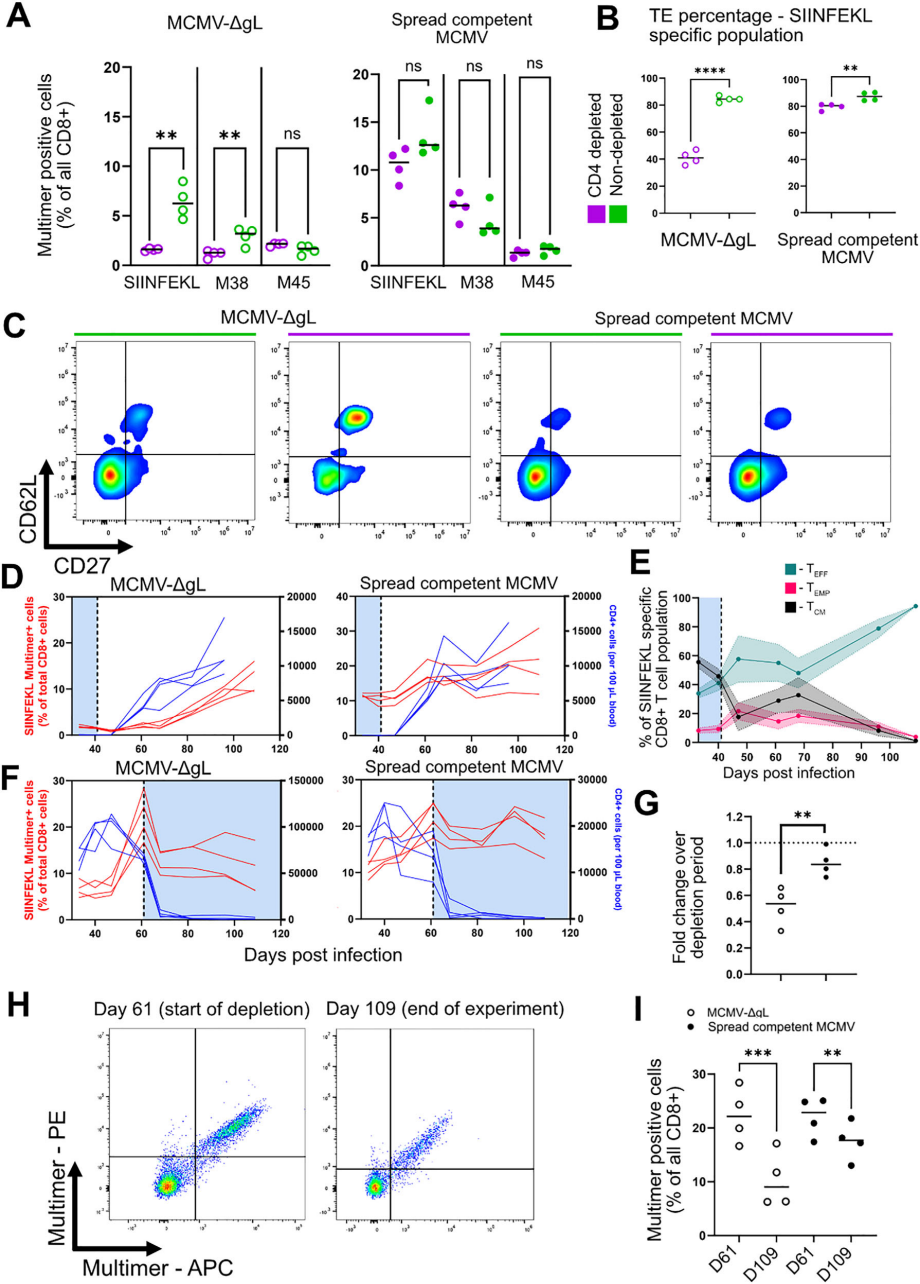
CD4 depletion in C57Bl/6 mice. (A) Day 40 after infection with spread‐defective MCMV (left panel). Here, mice in which CD4 cells are depleted show significantly smaller inflationary populations (SIINFEKL, M38) than non‐depleted mice. In mice infected with spread competent MCMV (right panel), CD4 depletion did not affect inflationary population size. (B) Effector differentiation of SIINFEKL‐specific population is lower in CD4‐depleted mice than in non‐depleted mice, especially in spread‐defective virus‐infected mice (top). (C) Exemplary FACS plots showing effector differentiation at day 40 post infection of SIINFEKL‐specific CD8^+^ T cells. A predominantly central memory phenotype is seen only in CD4‐depleted mice infected with spread‐defective virus. (D and F) CD4^+^ and SIINFEKL‐specific CD8^+^ T cell counts over time. Light blue shading indicates period of CD4 depletion injections. After release of depletion (D), the negligible SIINFEKL‐specific population in spread‐defective MCMV‐infected mice undergoes late expansion into an inflationary population. Late CD4 depletion (F) induces a decrease in the SIINFEKL population within the total CD8^+^ T cell population, especially in mice infected with spread‐defective virus. Percentage decrease over depletion period (day 61–109) is shown in (I), and fold decrease (also day 61–109) is shown in (G). (E) Effector differentiation status of SIINFEKL‐specific cells over time in spread‐defective virus‐infected mice. While predominantly central memory during the depletion period, the SIINFEKL‐specific population becomes predominantly effector differentiated after depletion is released. Differentiation defined by CD27 and CD62L expression: effector memory—CD27^−^CD62L^−^, T effector memory precursor—CD27^+^CD62L^−^, central memory—CD27^+^CD62L^+^. Graph shows mean with standard deviation indicated by shaded area. (H) Exemplary FACS plots showing decline of SIINFEKL‐specific population after CD4 depletion in spread‐defective virus‐infected mice. Difference between groups evaluated in (B and G) by unpaired Student's *t*‐test, differences in (I) evaluated by paired Student's *t*‐test. **p < 0.01, ***p < 0.001, ****p < 0.0001.

Having demonstrated CD4^+^ T cell support for effector differentiation in the Rag KO transfer model, we sought to confirm these findings in wild‐type mice. We modulated CD4^+^ T cell presence in C57BL/6 mice by administering the CD4‐specific antibody GK1.5 and monitored three endogenous MCMV‐specific T cell responses by serial blood sampling and multimer staining: two inflationary responses (M38 and SIINFEKL) and one classical response (M45). In the spread‐defective MCMV‐ΔgL‐infected mice, CD4 depletion markedly reduced both SIINFEKL and M38 response magnitudes (Figure 4A). This effect was not observed in the classical M45 response or in any response in mice infected with spread‐competent virus. As in the Rag KO transfer model, the absence of CD4^+^ T cells reduced effector differentiation of inflationary populations in MCMV‐ΔgL‐infected mice (Figure 4B,C), with this effect also observed to a lesser extent in spread‐competent infections. Conversely, M45‐specific cells showed less central memory differentiation in CD4‐depleted mice (Figure S5A).

In CD4‐depleted mice, GK1.5 injections were stopped 40 days post‐infection. In spread‐defective virus‐infected mice, recovery of peripheral blood CD4^+^ T cells was followed by late expansion of the SIINFEKL MI response (Figure 4D), which also developed greater effector differentiation over time (Figure 4E). Conversely, in mice without initial CD4 depletion, we induced CD4 depletion at day 61, at which point a large SIINFEKL MI population had become established (Figure 4F,H). In MCMV‐ΔgL‐infected mice, CD4 depletion coincided with a clear reduction in the SIINFEKL‐specific population within the CD8^+^ compartment, with this effect also observed to a lesser extent in spread‐competent MCMV‐infected mice (Figure 4G,I). Such a decrease was not seen in another experiment, monitoring SIINFEKL response without depletion over this timeframe (Figure S5B). The effector differentiated proportion of SIINFEKL‐specific T cells did not decrease over the course of CD4 depletion (Figure S5C).

### 2.5 Transcriptional Analysis Indicates Cytokine Signaling Differences Between Inflation Permissive and Nonpermissive Conditions

**FIGURE 5:**
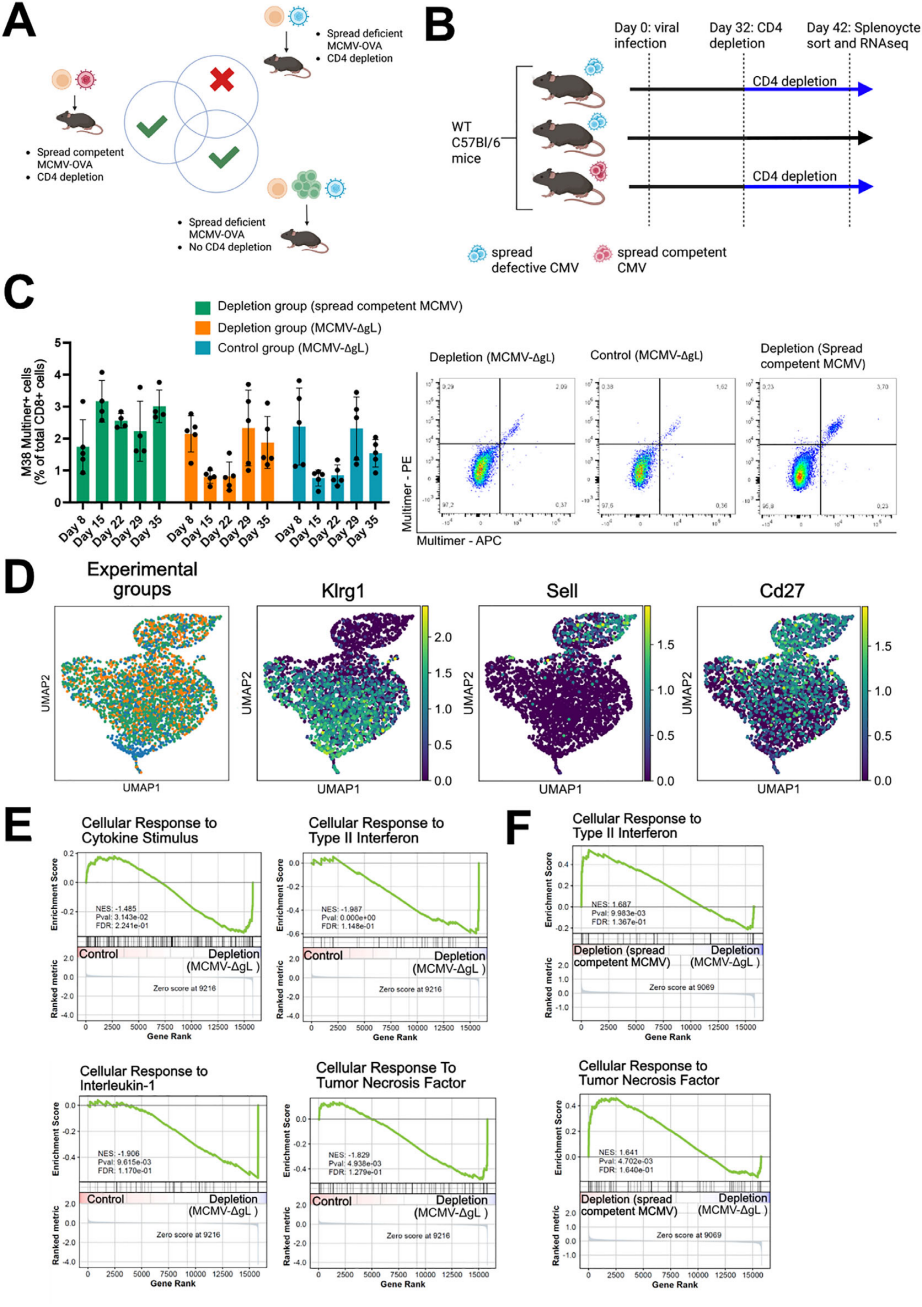
(A) Experimental concept. Memory inflation responses can develop during spread‐defective virus infection if CD4^+^ T cells are present, or in spread‐competent virus infection, even if CD4^+^ T cells are absent. If both spread competence and CD4^+^ T cells are absent, memory inflation does not occur. We sought to investigate factors underlying these distinct inflation‐permitting conditions. (B) Experimental design. Groups were set up corresponding to the three outlined conditions, three to four mice per group. CD4 depletion was initiated after inflationary growth of the M38 response was established. (C) M38 responses by group over time (left) with representative flow cytometry plots on day 35 (right). (D) UMAP visualization of scRNAseq data. Cells are colored by experimental group (left), Klrg1 expression (center‐left), CD62L (Sell) expression (center‐right) and Cd27 expression (right). (E) GSEA comparing CD4‐depleted versus non‐depleted groups in spread‐defective MCMV infection. CD4 depletion is associated with downregulation of cellular response to cytokine signaling, cellular response to type II interferon (IFNγ), cellular response to tumor necrosis factor, and cellular response to Interleukin 1. (F) Comparison of CD4‐depleted mice infected with spread‐defective versus spread‐competent MCMV. IL‐1 and type II interferon (IFNγ) responses are upregulated in spread‐competent MCMV infection.

Our experiments have so far shown that when CD4^+^ T cells are present, MI could develop during CMV infection even when the virus is spread defective, consistent with previous reports [24]. Conversely, MI could develop in response to spread‐competent CMV infection, even when CD4^+^ T cells were absent. However, in the absence of both CD4^+^ T cells and viral spread competency, IE2‐SIINFEKL and M38 responses failed to develop the effector differentiation characteristic of MI (Figure 5A). Cytokines are well‐established mediators of CD4^+^ T cell help for CD8^+^ T cell responses [25], and can also modulate CD8^+^ T cell responses when produced in the context of viral activity [26]. We were therefore particularly interested in cytokine signaling as a possible basis for the differences in MI responses we observed depending on the presence of T cell help and viral spread competence.

To identify molecular mediators that may facilitate MI under these distinct conditions, we performed single‐cell RNA sequencing of M38‐specific CD8^+^ T cells from mice representing all three scenarios (Figure 5B). We infected two groups of mice with spread‐defective virus (MCMV‐ΔgL) and allowed MI to become established through day 32 post‐infection (Figure 5C). At this timepoint, we depleted CD4^+^ T cells in one group while leaving the other undepleted. A third group was infected with a spread‐competent virus and underwent CD4^+^ depletion in parallel with one MCMV‐ΔgL group. One week after depletion, we isolated and sequenced M38‐specific CD8^+^ T cells from all three groups.

Single‐cell RNA sequencing revealed that cells predominantly expressed effector‐associated genes such as *Klrg1*, with a smaller fraction expressing CD62L and CD27, consistent with the known MI phenotype (Figure 5D). Cells from all groups formed a single cluster on the UMAP projection, indicating limited overall transcriptional state differences. We performed differential gene expression analysis between groups and used the ranked gene lists for gene set enrichment analysis with the GO_Biological_Process_2023 database. Processes were then filtered by FDR q‐value < 0.25, focusing on those involved in cytokine signaling. CD4 depletion in MCMV‐ΔgL‐infected mice led to downregulation of the cellular response to cytokine stimulus, and more specifically to downregulation of cellular responses to Type II Interferon (IFNγ), Tumor Necrosis Factor, and Interleukin‐1 (Figure 5E). Comparing the two CD4‐depleted groups, mice infected with spread‐competent virus showed upregulation of cellular responses to type II interferon and tumor necrosis factor (Figure 5F) compared to spread‐defective virus‐infected mice. Comparison between the two MI‐permissive groups (spread‐competent virus/CD4 depletion vs. spread‐defective virus/CD4 non‐depleted) yielded no cytokine signature differences meeting our threshold criteria.

## 3 Discussion

CD4^+^ T cells have a long‐described role in supporting CD8^+^ T cell responses [25], including MI [15, 17]. However, how CD4^+^ T cells support MI is not understood. In this study, we set out to investigate how CD4^+^ T cells facilitate CD8^+^ MI in CMV infection, focusing on a spread‐defective CMV as a model of spread‐defective CMV vaccination vectors.

We observed a failure of transferred CD8^+^ T cells to develop a MI response in Rag KO mice, consistent with previous reports in CD4^+^ T cell deficient mice. Specifically, in this system, we observed an as‐yet unreported failure of inflationary‐epitope specific CD8^+^ T cells to form predominantly effector differentiated populations, a defining characteristic of MI. In contrast, these cells formed predominantly central memory phenotype populations. Walton et al. and Snyder et al. did not observe this effect on effector differentiation [15, 17]. However, experiments in these studies used spread competent MCMV, for which we saw a much smaller effect on differentiation than with spread‐defective MCMV. Furthermore, these papers primarily used knockout mouse models to assess CD4^+^ T cell absence, which have additional deviations from WT mice that may influence MI responses. CD4 knockout mice develop MHCII‐restricted CD8^+^ T cells [27], as well as CD4^−^CD8^−^ T cells with helper functions [28], while MHCII knockout mice show impaired maintenance of central memory T cells [29]. An additional difference between our experiments and Walton et al. is the method of constraining viral activity. Walton et al. administered famciclovir starting at day 30 post infection, whereas MCMV‐ΔgL is immediately spread defective once it has infected host cells. We observed impaired effector differentiation only when both spread deficiency and CD4^+^ T cell absence were present from the start of infection.

Interestingly, although we observed that CD4^+^ T cells absence impairs effector differentiation in MI, the opposite was seen in the classical M45 response (Figure S5A), consistent with the described role of T cell help in establishing CD8^+^ T cell memory [14, 30]. Thus, although failed MI responses follow numerical kinetics similar to classical memory responses, they remain fundamentally distinct, presumably due to chronic antigen presentation from latently infected cells. This may also be reflected in our Rag KO transfer experiments, where we observed a trend toward central memory population growth outpacing homeostatic proliferation in spread‐defective MCMV‐infected mice.

CD4^+^ T cells support CD8^+^ T cell responses through multiple described mechanisms, including well‐characterized roles during the priming stage of CD8^+^ T cell activation [31, 32], although continuous cytokine support during chronic LCMV infection [33] and memory maintenance [34] have also been demonstrated. Through provision of CD4^+^ T cells to deficient mice, we show that this differentiation support can be mediated outside of the priming window at the start of infection. Furthermore, by CD4 depletion in mice with established MI populations, we demonstrate that continuous CD4^+^ T cell support is involved in the maintenance of MI. Within the depletion period, most of the decrease in SIINFEKL‐specific CD8^+^ T cells occurred shortly after CD4 depletion, consistent with the possibility that subsets within the MI population have differing levels of CD4^+^ T cell support requirements. Interestingly, we observed a 0.5‐fold reduction over the 48‐day depletion period (Figure 4G), which corresponds well to a 45‐ to 60‐day half‐life of inflationary cells described in literature [35].

Previous research on the role of CD4^+^ T cells in MI has primarily focused on knockout models that preclude investigation of temporal dynamics, although there are limited reports using antibody‐mediated CD4^+^ depletion—late depletion from 3 weeks post‐infection in Snyder et al. appeared to weaken the IE3 response, consistent with our results, though lack of longitudinal measurements prevents determining whether this reflects decline of established MI or early arrest of a growing response.

The apparent decline in SIINFEKL‐specific T cells we observed over the late depletion period might be alternatively explained by homeostatic forces [36]. Specifically, the rapid loss of the CD4^+^ T cell compartment may induce a general proliferation of CD8^+^ T cells, such that the proportion of SIINFEKL‐specific CD8^+^ T cells is reduced even if the population is stable. However, comparable declines were not observed in M38‐ and M45‐specific populations (Figure S5D). A reduction in the SIINFEKL population was also seen in mice infected with spread‐competent CMV, which can induce inflationary SIINFEKL responses even in the absence of T cell help, although this was significantly smaller in magnitude than that observed in spread‐defective MCMV‐infected mice (Figure 4G). We did not observe a significant decline in the M38 response over the late depletion period (Figure S5D). This may reflect the differential sensitivity of MI response to the absence of CD4^+^ T cell help. Nonetheless, the M38 population effector differentiation was impaired in CD4‐depleted mice. Additionally, in our model, the M38 response is somewhat suppressed by competition with the highly immunogenic SIINFEKL response [37], resulting in lower absolute numbers that may obscure subtle declines following CD4 depletion.

Our transfer of CD4^+^ T cells into Rag KO mice improved the expansion of CD8^+^ T cell effector cells. However, this effect was transient in our early transfer experiment, when CD4^+^ T cells should be able to support CD8^+^ T cell priming. In line with our late depletion data in WT mice, this suggests that priming support is, at least, insufficient by itself to maintain a response with a full MI phenotype. The limited ability of transferred CD4^+^ T cells to sustain effector populations in Rag KO mice may reflect the transferred dose of 1.2 × 10^6^ cells, which is considerably smaller than physiological CD4^+^ T cell populations and consequently provides limited repertoire diversity and cytokine‐producing capacity. Importantly, while our data support the relevance of T cell help outside the priming period of CD8^+^ T cell activation, we cannot exclude that support during priming is important for MI. Indeed, inflationary cells primed in MHCII KO mice show weaker responses when transferred into WT mice than those primed in WT donors [15].

A key question that follows from our observations is how CD4^+^ T cells can provide this priming‐independent support, and why inflationary responses to spread competent MCMV are less reliant on T cell help. On this latter point, it is interesting to note that dependence on CD4^+^ T cell help, at least for the primary CD8^+^ T cell response, is generally diminished in high inflammatory settings [38], in which the requirement for help can be circumvented for instance by non‐CD4^+^ T cell‐derived Type 1 IFN [39]. Such mechanisms have not been demonstrated in the context of MI; however, Walton et al. speculated that persistent CMV activity in CD4^+^ T cell‐deficient mice can provide additional antigen stimulation to inflationary T cells via antigen‐loaded APCs [17]. To determine how CD4^+^ T cells support MI, previous approaches have focused on knockout models of plausible signaling molecules including IL‐21 2018 [19], IL‐2 [17], CD40L and OX40 [15]. While these knockouts have clear effects on MI responses, such organism‐wide modifications make it difficult to attribute effects specifically to CD4^+^ T helper cell signaling. IL‐2, for instance may be produced autologously by CD8^+^ T cells themselves [40]. To identify possible mediators of continuous CD4^+^ T cell support in MI, we inferred cytokine signals from transcriptomic data of M38‐specific CD8^+^ T cells in CD4‐depleted and non‐depleted mice. GSEA analysis indicated several candidate cytokines that may mediate CD4^+^ T cell help during spread‐defective virus infection, namely IFNγ, TNF, and IL‐1. IFNγ and TNF are especially plausible mediators, as both can be produced by CMV‐specific CD4^+^ T cells during infection [41, 42]. These are, however, both pleiotropic cytokines with complex, context dependent effects. IFNγ and TNF have both been implicated in promoting cell death during the contraction phase of T cell responses [43, 44], but have also been shown to promote T cell proliferation [45, 46, 47]. Furthermore, IFNγ can enhance T cell motility, survival, and cytotoxicity (Bhat et al. 2017 [48]). Interestingly, in MHCII KO mice, the deficiency in maintaining central memory populations (described above) has been attributed to the production of IFNγ by colonic T cell populations in this mouse line [29], underlining the potential relevance of T cell‐derived IFNγ to subsequent T cell differentiation. IFNγ and TNF response signatures were also elevated in the spread‐competent virus group despite CD4 depletion, indicating that viral activity may induce sufficient levels of these cytokines to support inflation in the absence of production from CD4^+^ T cells. It is important to note, however, that these transcriptional signatures could alternatively reflect the MI effector state itself, which may share gene expression patterns with IFNγ/TNF‐stimulated cells independent of actual cytokine exposure. Furthermore, even if these signatures reflect actual cytokine exposure, this itself is not sufficient to prove that the cytokines in question are relevant for driving the MI phenotype. These signatures may nonetheless be informative to the question of why inflationary responses do not develop against spread‐defective virus in the CD4^+^ T cell deficient mouse. The low IFNγ signature in these mice, for example, may be indicative of a general low IFNγ production in inflationary cells themselves, and a consequent failure to maintain latency pool, since interferon signaling is important for maintaining CMV in latency [49]. This explanation is consistent with previous work showing knockout of STAT1, which relays IFNγ signaling [50], produces a system in which the latent reservoir of spread‐defective CMV is not maintained, likely due to an inability of CD8^+^ T cells to repress lytic activity [51]. Accordingly, our data could be explained by a requirement of CD4^+^ T cell support for MI cells to produce sufficient IFNγ to maintain latency in spread‐defective CMV infection. In the absence of CD4^+^ T cells, viral spread competence could adequately stimulate MI cells to produce sufficient IFNγ. In spread‐defective CMV‐infected mice, this loss of latent reservoir in the absence of CD4^+^ T cells would prevent the development of MI responses if CD4^+^ T cells are present at a late timepoint. However, our data show that such late MI responses reliably develop in both the Rag KO transfer system, and upon release of depletion in WT mice, demonstrating the latent reservoir remains intact.

The final potential cytokine mediator from our GSEA analysis, IL‐1, can also support expansion and effector functions of CD8^+^ T cells [52], particularly in viral infection [53, 54]. However, IL‐1 is not produced by CD4^+^ T cells but is instead produced mainly by innate immune cells. Nonetheless, CD4^+^ T cells can enhance IL‐1 production from dendritic cells via CD40‐CD40L interaction [55], which may represent an indirect mechanism by which CD4^+^ T cells can support MI through IL‐1. Ultimately, the relevance of these candidate mediators of CD4^+^ T cell support of MI will require validation through more targeted experimental approaches.

An additional question is whether CD4^+^ T cell specificity for CMV antigens is required for supporting MI. The relevance of CD4^+^ T cell specificity varies by support mechanism; while CD4^+^ T cell cross‐priming via dendritic cells is antigen‐dependent [56], CD4^+^ T cells can also support maintenance of memory CD8^+^ T cells through antigen‐independent tonic signaling [34]. We attempted to address this question by transferring SMARTA cells—CD4^+^ T cells specific for the non‐CMV peptide gp33 [57]—into Rag KO mice. Unlike WT CD4^+^ T cell transfer, SMARTA transfer did not significantly improve effector differentiation of inflationary CD8^+^ T cells. However, this result is difficult to interpret, as SMARTA cells expanded poorly following transfer compared to WT cells, likely due to antigen‐driven expansion of CMV‐specific clones within the polyclonal WT population. The resulting low numbers of SMARTA cells may have been insufficient to provide detectable nonspecific support. To address this confounding factor, future work could include transfer of CD8^+^ T cells into SMARTA or WT host mice, where CD4^+^ T cell populations are more stably maintained through normal homeostasis.

An important caveat of this study is that inflationary responses show variable properties depending on the epitope. Besides the differential CD4^+^ T cell dependence described above, inflationary responses also vary by kinetics [58], sensitivity to dendritic cell deficiency [59], and gene expression patterns of their respective epitopes during latency [60]. Mechanisms relevant for a given MI response may therefore not apply uniformly across all inflationary epitopes. Further investigation of additional MI responses will be necessary to determine the generalizability of CD4^+^ T cell support mechanisms.

In conclusion, our study clarifies the nature of CD4^+^ T cell help in generating MI responses to spread‐defective CMV. We demonstrate that in this context, CD4^+^ T cell help is required not only for the expansion of MI, but also for their effector differentiation. Our data suggest a continuous support mechanism beyond the priming stage, likely mediated by cytokines. Maintenance of large effector populations is an attractive quality for cell therapies, and understanding how MI develops could inform design strategies to improve persistence and functionality. Our findings are particularly relevant to spread‐defective CMV and adenovirus vaccination strategies that seek to harness MI for durable responses.

## 4 Materials and Methods

### 4.1 Mice

C57Bl/6JOlaHsd (females, 6–8 weeks old) were purchased from Envigo. Female 6‐ to 24‐week‐old Rag KO mice (B6.129S7‐Rag1tm1Mom/J), Rag KO congenic matrix mice (B6.129S7‐Rag1tm1Mom/J x (B6.SJL‐PtprcaPepcb/BoyJB6.PL‐Thy1a/CyJ), OT‐I mice (C57BL/6‐g(TcraTcrb)1100 Mjb/J), and SMARTA mice (B6;D2‐Tg(TcrLCMV)Aox/J) were originally obtained from The Jackson Laboratory and bred under specific pathogen‐free conditions at our mouse facility at the Technical University of Munich.

### 4.2 Sample Collection and Preparation

Blood samples were collected by lancet puncture of the superficial temporal vein (cheek puncture) into 1.5‐mL Eppendorf tubes with 2 µL of Heparin (Ratiopharm) to prevent coagulation. Erythrocytes were lysed by incubation of the sample in Ammonium‐Chloride‐Tris (ACT) buffer. For spleen samples, mice were euthanized by cervical dislocation. Spleens were then removed and mashed through a 70‐µm strainer into ACT buffer for erythrocyte lysis. After lysis, cells were washed in FACS buffer (PBS with 0.5% BSA and 2 mM EDTA) before proceeding to staining.

### 4.3 Flow Cytometry

For surface antibody staining, cells were incubated with respective antibody panels (diluted in FACS buffer) for 15 min at 4°C in the dark. Antibodies used are listed in Table S1. Cells were then washed with FACS buffer. Cells were acquired on CytoFLEX S or CytoFLEX LX flow cytometers (Beckman Coulter), in FACS buffer containing 1:100 propidium iodide (PI) as a live/dead stain. Analysis was performed with FlowJo software (FlowJo LLC).

### 4.4 Multimer Staining

Biotinylated peptide–MHC multimers were refolded as previously described [61]. For multimerization, for every 5 × 10^6^ cells to be stained, 0.4 µg of biotinylated pMHC I was preincubated with 0.5 µg of streptavidin‐PE or streptavidin‐APC for at least 40 min at 4°C in the dark. Cells were stained with PE multimer mix for 15 min, then with both PE and APC multimers for 30 min, at 4°C in the dark. Cells were then washed in FACS buffer and processed for surface antibody staining and flow cytometry as described above.

### 4.5 T Cell Transfers

To collect CD8^+^ T cells, blood samples were taken from OT‐I or T cell receptor retrogenic mice. After erythrocyte lysis and antibody staining, CD8^+^, congenic marker‐positive, and CD44 low/negative cells were sorted by FACS onto recipient‐matched feeder cells in 200 µL FCS in a V‐bottom plate. In the case of retrogenic mice, cell sorting included selection for cells with a fluorescent reporter molecule (BFP or CFP). Cells were then administered to recipient mice, one well content per mouse, by intraperitoneal injection.

For CD4^+^ T cell transfers, spleens were collected from donor mice and processed as described above. Cells were sorted by FACS, firstly by gating out CD19^+^ and CD8^+^ cells, then by selecting CD3^+^ and CD4^+^ double‐positive cells. Sorted cells were re‐suspended in FCS and administered to recipient mice in 200 µL doses by intraperitoneal injection.

### 4.6 CD4 Depletion

A monoclonal antibody against murine CD4, GK1.5 (BioXcell), was administered to mice by intraperitoneal injection. Each dose contained 100 µg of antibody diluted in 200 µL PBS. To establish CD4 depletion, doses were administered on three consecutive days. For maintenance of CD4 depletion, doses were administered once per week.

### 4.7 Viruses

The spread competent MCMV‐IE2‐SIINFEKL virus and spread‐defective MCMV‐ΔgL‐IE2‐SIINFEKL virus were generated by en passant mutagenesis from the BAC‐derived MCMV pSM3fr‐MCK‐2fl clone 3.3, as previously described [22]. MCMV‐IE2‐SIINFEKL stocks were isolated from the salivary gland tissue of infected Balb/c mice. MCMV‐ΔgL‐IE2‐SIINFEKL stocks were produced by cell culture with gL‐3T3 packaging cells.

### 4.8 Infection

Viruses were administered by intraperitoneal injection in doses of 200 µL PBS. For spread competent MCMV‐IE2‐SIINFEKL, each administered dose contained 500 Plaque‐Forming Units (PFU). For the spread‐defective MCMV‐ ΔgL‐IE2‐SIINFEKL virus, doses ranged from 1 × 10^5^ to 1 × 10^6^ PFU, and are indicated in figure legends. MVA‐Ova was administered at a dose of 5 × 10^6^ PFU per mouse.

### 4.9 Functionality Assay

Splenocytes were isolated and divided between wells of a 96‐well U‐bottom plate, and incubated at 37°C with a range of SIINFEKL peptide concentrations (10^−6^ to 10^−13 ^M) in cRPMI. After 1 h incubation, Golgi Plug (BD Bioscience) (2 µL per well) was added, followed by a further 4 h of incubation. After incubation, cells were washed in FACS buffer, and stained with ethidium monoazide (EMA, 1:1000) and Fc‐block (1:400) under bright light at 4°C for 20 min. Cells were washed in FACS buffer again, and a surface antibody stain was applied as described. Next, cells were fixed in 2% RotiFix (Carl Roth) for 40 min, and washed in Permwash buffer (BD Bioscience). An intracellular stain for cytokines was then performed in Permwash buffer. Cells were then washed in FACS buffer and acquired at a flow cytometer. For calculating EC50 values, maximal cytokine positive percentages across the peptide concentration gradient were normalized to 100%, and a nonlinear curve was fitted to the normalized data.

### 4.10 Generation of Retrogenic Mice

Bone marrow was isolated from femur and tibia of Rag KO congenic marker mice. Bone marrow was washed in FACS buffer, resuspended into a single cell suspension, and stained for Sca‐1, CD19 and CD3, and PI for live/dead discrimination. Hematopoietic stem cells (HSCs) were identified as Sca‐1^+^, CD19, and CD3 double negative. HSCs were sorted by FACS and incubated in cDMEM containing murine IL‐3 (20 ng/mL), IL‐6 (50 ng/mL), and SCF (50 ng/mL) for 3–4 days at approx. 250,000 cells per mL. For retroviral transduction, supernatant from retroviral vector (with fluorescent reporter and TCR) transfected Platinum‐E cells was centrifuged in RetroNectin (Takara)‐coated tissue culture untreated 48‐well plates. Supernatant was partially removed, leaving 200 µL, after which 200 µL of stem cell suspension was added to each well in cDMEM with double cytokine concentrations. Stem cells were spinoculated and rested for 2 days in culture before transfer into irradiated C57Bl/6 recipient mice.

### 4.11 Tissue Culture

Platinum‐E cells for retrovirus production were cultured in complete DMEM (Life Technologies), containing 10% Fetal Calf Serum, 0.025% L‐glutamine, 0.1% HEPES, 0.001% gentamycin, and 0.002% streptomycin.

### 4.12 Statistics

Statistical analysis was performed with GraphPad PRISM software. Statistical tests and graphical representation used are indicated in the figure legends.

### 4.13 Single‐Cell RNA Sequencing

Splenocytes were isolated from sacrificed mice, and stained with hashtag antibodies according to the sample. Specifically, hashtag antibodies against CD45 and MHC I were pre‐spun at 14,000G for 10 min before use, then incubated with cells at a 1:50 dilution. Cells were then washed five times with FACS buffer to remove unbound hashtag antibody. Cells were then stained with multimer and antibodies to allow FACS isolation of M38‐specific CD8^+^ T cells. After sorting, cells were added to a 10x chip and processed according to the manufacturer's protocol (Chromium next GEM Single Cell VDJ V1.1 with Feature Barcode, Rev D). Libraries were sequenced on an Illumina NovaSeq X Plus (PE150), generating a mean depth of 40K reads/cells. Reads were aligned to the mm10‐2020‐A reference genome using Cell Ranger 7.0.0 (10x Genomics). HTO demultiplexing was performed using HashSolo [62]. Downstream analysis was performed in Python 3.11 using scanpy 1.11.2. Cells were filtered by minimum 200 genes, UMI counts between 2000 and 30,000, mitochondrial gene fraction below 10%, and a minimum of 750 genes expressed.

### 4.14 Cytokine Signaling Inference

Differentially expressed genes were identified using diffxpy with thresholds of |log_2_FC| ≥ 0.25 and adjusted *p* < 0.05. Significant genes were ranked by log_2_ fold‐change and analyzed using GSEApy (pre‐ranked mode). Gene ontology sets from GO_Biological_Process_2023 were used as the reference database. Enrichment significance was assessed from 1000 permutations, and gene sets with FDR *q*‐value ≤ 0.25 were considered significant. Gene sets meeting this criterion are listed in Table S2. From these, gene sets related to cytokine signaling were identified based on the presence of cytokine‐associated keywords (“Interleukin,” “Interferon,” “Cytokine,” “Chemokine,” “Tumor Necrosis Factor”) and by manual curation to include additional cytokine‐relevant processes.

## Author Contributions

J.B. designed and performed experiments, conducted formal analysis of the data, and wrote the manuscript. M.T.W. and J.B. developed and performed software analyses. Y.K. developed a spread‐defective virus and produced virus stocks. All authors read and approved the manuscript. D.H.B conceptualized the study and acquired most of the funding. D.H.B., M.P.P., V.R.B., and L.C‐S. supervised the study and administered the project. AI tools were used to assist with language editing and sentence restructuring of the manuscript text. Figures were produced with the use of BioRender.com

## Funding

This research was supported by SFB 1054 (TP‐B09), SFB‐TRR 338/1 2021‐452881907 (A01), and SFB‐1371 395357507 (P04).

Open Access funding enabled and organized by Projekt DEAL.

## Ethics Statement

All animal experiments were approved by the district government of upper Bavaria (Department 5—Environment, Health and Consumer Protection; vote 55.2‐2532.Vet_02‐21‐155, vote 55.2‐2532.Vet_02‐21‐89 and vote 55.2‐2532.Vet_02‐18‐168).

## Conflicts of Interest

D.H.B. and M.P.P. are co‐founders of Match Medicines GmbH. The other authors declare no conflicts of interest.

## Supporting information




**Supporting File 1**: eji70225‐sup‐0001‐SuppMat.pdf.

**Supporting File 2**: eji70225‐sup‐0002‐TableS2.xlsx.

## Data Availability

The data that support the findings of this study are available from the corresponding author upon reasonable request.
