# Supplementary material for: The Memory Inflation Response Against Spread‐Defective CMV Requires Priming‐Independent CD4+ T Cell Help
Source: Eur J Immunol. 2026 Jun 17;56(6):e70225. doi: 10.1002/eji.70225 (PMC13273925; doi:10.1002/eji.70225)
Supplement: Supplementary file 1 — Supporting File 1: eji70225‐sup‐0001‐SuppMat.pdf. [file EJI-56-e70225-s001.pdf]

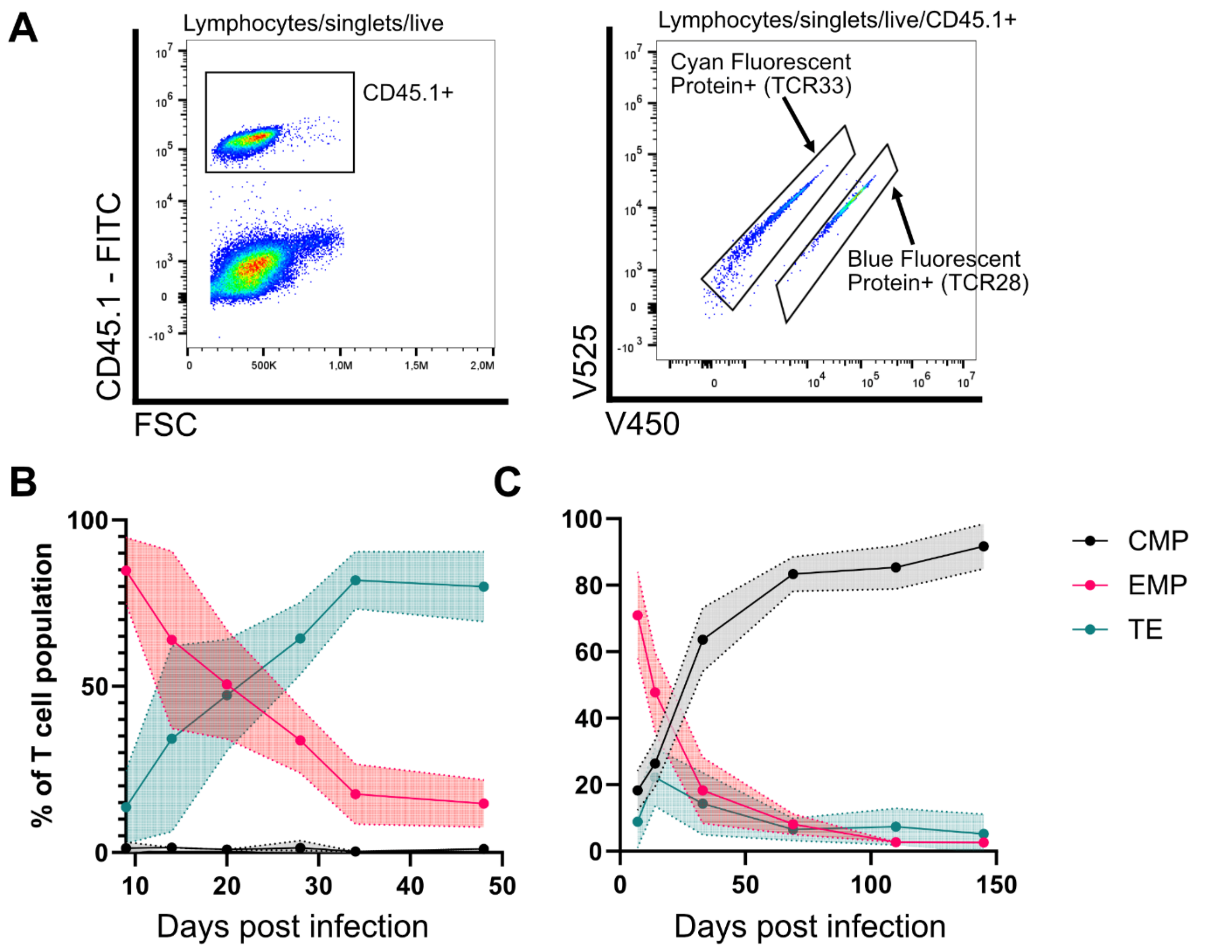

Figure S1

(A) Tracking of transferred T cell populations, representative plots. Left panel – transferred cells derive from retrogenic mice, generated with bone marrow from mice expressing congenic marker CD45.1. This allows easy discrimination from endogenous cells of recipient mice, which are CD45.2+/+. Right – transferred T cell populations are distinguished from one another by expression of fluorescent reporters. Higher affinity TCR33, Cyan Fluorescent Protein. Lower affinity TCR28, Blue Fluorescent Protein. (B, C) Data for lower affinity T cell population, TCR28. Shaded area indicates standard deviation. (B) T cell effector differentiation over time in response to host infection by spread competent MCMV-IE2-SIINFEKL infection (500 PFU). Typical of memory inflation responses, T effector cells became predominant in the population. (C) T cell differentiation over time in response to host infection by spread defective MCMV-ΔgL-IE2-SIINFEKL (1 x 10^5 PFU), showing a lack of effector differentiation, and predominance of CM cells over time. CM – Central Memory, EMP – Effector Memory Precursor, TE – T Effector.

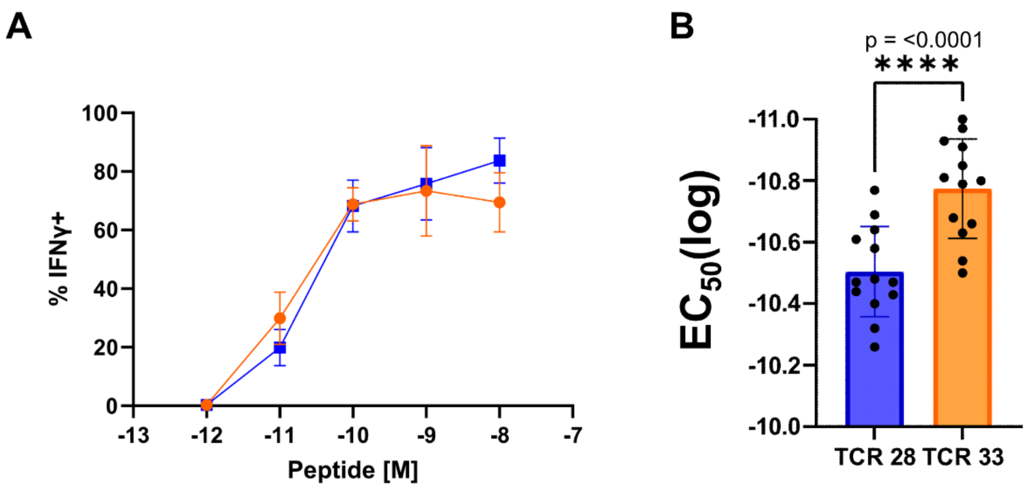

Figure S2

Functionality of transferred CD8+ T cell populations in MCMV-ΔgL-IE2-SIINFEKL infected Rag KO mice. Error bars indicate standard deviation. (A) IFN-γ response of peptide stimulated cells ex vivo. (B) Calculated EC50 values from IFN-γ response. Statistical evaluation by paired student's t test, ****p<0.0001

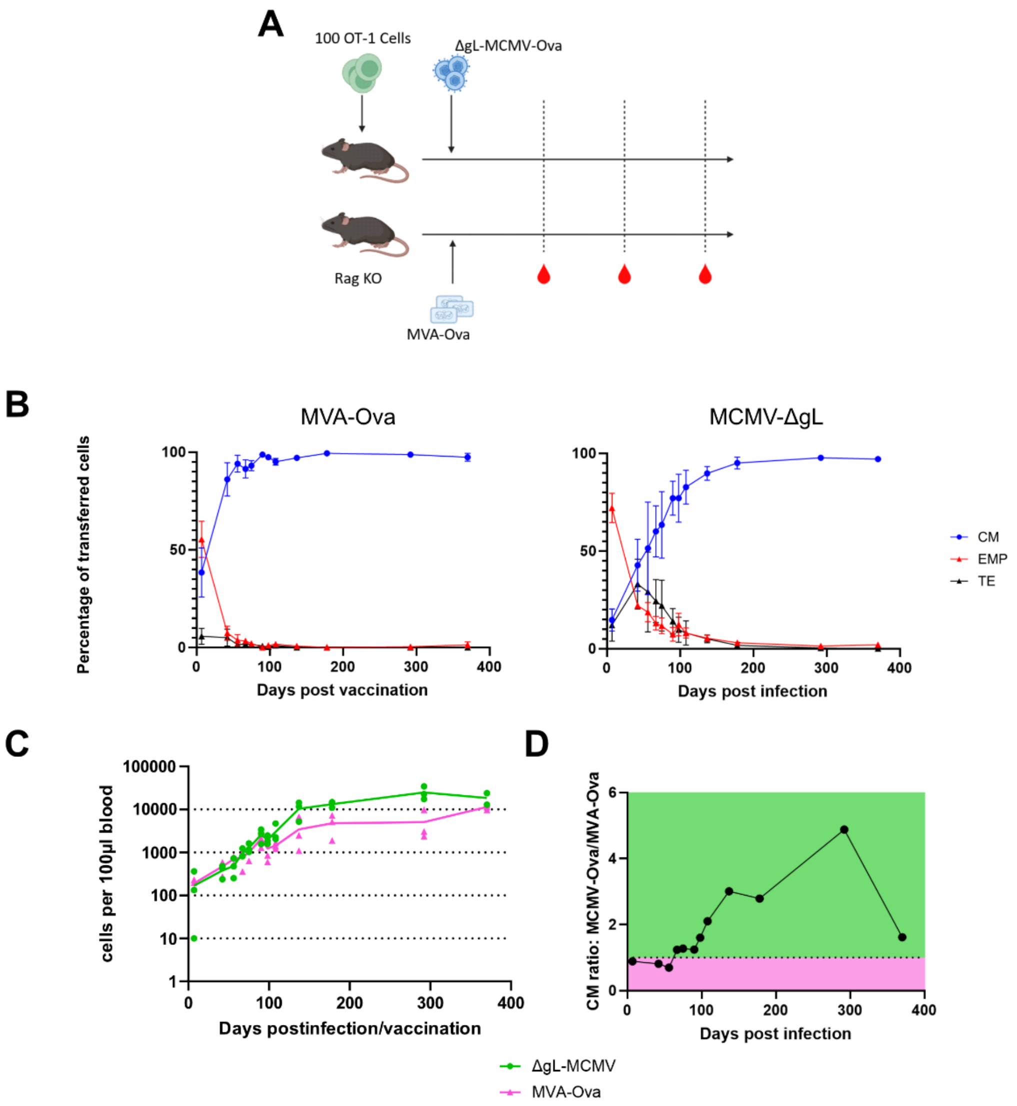

Figure S3

Response of transferred CD8⁺ T cells in Rag KO mice to attenuated virus with acute or chronic stimulation. (A) Experimental overview. Mice are infected with MVA-Ova or MCMV-ΔgL-IE2-SIINFEKL, and T cell response monitored by serial blood sampling. (B) In both groups, CD8⁺ T cells form predominantly central memory populations over time. Error bars indicate standard deviation. CM – Central Memory, EMP – Effector Memory Precursor, TE – T Effector. (C) Central memory populations show continuous growth long after initial infection. Differences between groups were evaluated by two-sided Welch's t-tests with Benjamini-Hochberg correction across all timepoints. $p>0.05$ at all timepoints. (D) Ratio of central memory cell concentration in blood (cells per 100 µL) over time, between groups. Green shading – more central memory cells in MCMV-ΔgL infected mice, pink shading – more central memory cells in MVA-Ova vaccinated mice. While central memory populations are initially comparable between groups, MCMV-ΔgL-IE2-SIINFEKL infected mice develop larger central memory populations over time.

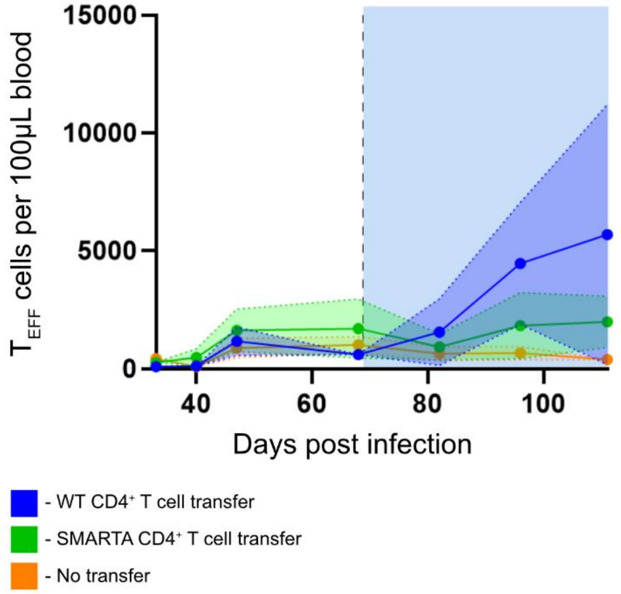

Figure S4

Absolute counts of effector differentiated CD8+ T cells (CD27$^-$CD62L$^-$) in peripheral blood. Light blue shading indicates time after CD4$^+$ T cell transfer. Differences between WT and no transfer groups were evaluated by two-sided Welch's t-tests with Benjamini-Hochberg correction across all timepoints. p>0.05 at all timepoints.

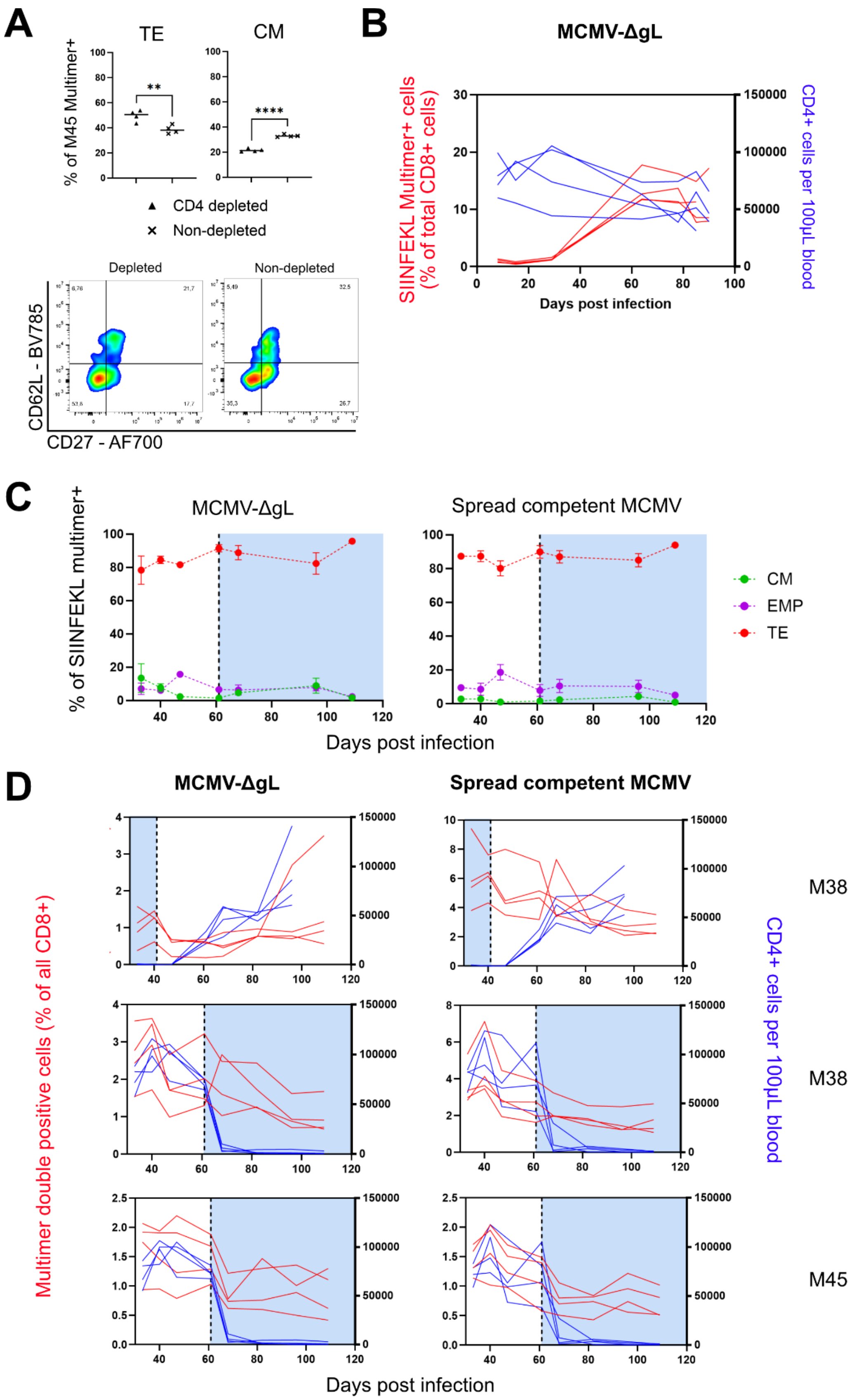

Figure S5

Endogenous MCMV T cell responses in MCMV infected C57Bl/6 mice. (A) At day 40 post infection, in MCMV-ΔgL-IE2-SIINFEKL infected mice, M45 specific cells in CD4

depleted mice show greater effector differentiation, and less central memory differentiation, contrary to the effect we see in cells specific for inflationary epitopes. Statistical evaluation by unpaired student's t test, **p<0.01, ****p<0.0001 (B) SIINFEKL response observed over 90 day non-CD4 depletion conditions in a separate experiment, following infection with 1 x 10^5 PFU MCMV-ΔgL-IE2-SIINFEKL. (C) Differentiation phenotype of SIINFEKL specific $CD8^+$ T cells over time. Predominantly effector differentiation is maintained throughout the late depletion period. Error bars indicate standard deviation. (D) Endogenous M38 and M45 specific responses, and $CD4^+$ T cells counts, over time. CD4 depletion period in (C) and (D) is represented by blue shaded area.

Supplementary Table 1: antibodies used (FACS and depletion)

Antibody	Source	Identifier	Dilution
aCD19 PE/Dazzle 594	BioLegend	115553, RRID:AB_2564000	300
aCD27 AlexaFluor 700	BioLegend	124240, RRID:AB_2810383	200
aCD3 PE/Cyanine7	BioLegend	100320, RRID:AB_312685	300
aCD3 BUV395	BD	569614, RRID:AB_3095858	100
aCD3 PE/Dazzle 594	BioLegend	100348, RRID:AB_2564029	300
aCD4 PE/Dazzle 594	BioLegend	100566, RRID:AB_2563685	300
aCD4 Pacific Blue	BioLegend	100531, RRID:AB_493374	100
aCD44 APC	BioLegend	103012, RRID:AB_312963	300
aCD45.1 FITC	BioLegend	110706, RRID:AB_313495	200
aCD45.1 PE	BioLegend	110708, RRID:AB_313497	300
aCD45.1 Pacific Blue	BioLegend	110722, RRID:AB_492866	100
aCD45.2 Pacific Blue	BioLegend	109820, RRID:AB_492872	100
aCD45.2 APC	BioLegend	109814, RRID:AB_389211	100
aCD62L BV785	BioLegend	104440, RRID:AB_2629685	100
aCD8 FITC	BioLegend	100706, RRID:AB_312745	200
aCD8 PE	BioLegend	100708, RRID:AB_312747	250
aCD8 PE/Cyanine7	BioLegend	100722, RRID:AB_312761	300
aCD8 Pacific Orange	Thermo Fisher Scientific	MCD0830, RRID:AB_10376311	50
aCD8 BUV785	BioLegend	100750, RRID:AB_2562610	200
aIFNγ APC	LifeTech	17-7311-82, RRID:AB_469504	400
aIL2 PE	BioLegend	503808, RRID:AB_315302	200

Antibody	Source	Identifier	Dilution
aKLRG1 PE/Cyanine7	BioLegend	138416, RRID:AB_2561736	100
aKLRG1 PE	BioLegend	138408, RRID:AB_10574313	100
aTNFa PE/Cyanine7	BioLegend	506324, RRID:AB_2256076	100
aSca1 PE	BioLegend	108107, RRID: AB_313344	100
aCD16/32 purified (Fc Block)	BioLegend	101301, RRID: AB_312801	400
TotalSeq-C0301 anti-mouse Hashtag 1	BioLegend	155861, RRID: AB_2800693	-
TotalSeq-C0302 anti-mouse Hashtag 2	BioLegend	155863, RRID: AB_2800694	-
TotalSeq-C0303 anti-mouse Hashtag 3	BioLegend	155865, RRID: AB_2800695	-
TotalSeq-C0304 anti-mouse Hashtag 4	BioLegend	155867, RRID: AB_2800696	-
TotalSeq-C0305 anti-mouse Hashtag 5	BioLegend	155869, RRID: AB_2800697	-
TotalSeq-C0306 anti-mouse Hashtag 6	BioLegend	155871, RRID: AB_2819910	-
TotalSeq-C0307 anti-mouse Hashtag 7	BioLegend	155873, RRID: AB_2819911	-
TotalSeq-C0308 anti-mouse Hashtag 8	BioLegend	155875, RRID: AB_2819912	-
TotalSeq-C0309 anti-mouse Hashtag 9	BioLegend	155877, RRID: AB_2819913	-
TotalSeq-C0310 anti-mouse Hashtag 10	BioLegend	155879, RRID: AB_2819914	-
TotalSeq-C0310 anti-mouse Hashtag 11	BioLegend	155881, RRID:AB_2924482	-
TotalSeq-C0310 anti-mouse Hashtag 12	BioLegend	155883, RRID:AB_2924483	-
TotalSeq-C0310 anti-mouse Hashtag 13	BioLegend	155885, RRID:AB_2922483	-
TotalSeq-C0310 anti-mouse Hashtag 14	BioLegend	155887, RRID:AB_2922484	-
InVivoMAb anti-mouse CD4	BioXcell	BE0003-1, RRID:AB_1107636	-
aStreptavidin PE	LifeTech	12-4317-87	-
aStreptavidin APC	LifeTech	17-4317-82	-

Antibody	Source	Identifier	Dilution
**Multimer**			
H2-Kb/mβ2m/Ova 257-264 Streptavidin-PE/APC	In house		
H2-Kb/mβ2m/M38 316-323 Streptavidin-PE/APC	In house		
H2-Db /M45 985-993/mß2m-PE/APC	In house		

Supplementary Table 2: Gene Set Enrichment Analysis results for pairwise comparisons described in Figure 5. This dataset is provided as a separate spreadsheet file (Supplementary_Table_2.xlsx).
